# 2-[5-(2-Methyl­phen­yl)-3-(2-methyl­styryl)-4,5-di­hydro-1*H*-pyrazol-1-yl]-6-(thio­phen-2-yl)-4-(tri­fluoro­meth­yl)pyrimidine chloro­form monosolvate

**DOI:** 10.1107/S160053681401321X

**Published:** 2014-06-18

**Authors:** Alex Fabiani Claro Flores, Darlene Correia Flores, Juliano Rosa de Menezes Vicenti, Patrick Teixeira Campos

**Affiliations:** a Escola de Química e Alimentos, Universidade Federal do Rio Grande, Av. Itália, km 08, Campus Carreiros, 96203-900 Rio Grande, RS, Brazil; b Instituto Federal Farroupilha, Campus Júlio de Castilhos, CEP 98130-000, Júlio, de Castilhos, RS, Brazil

## Abstract

In the crystal structure of the title compound, C_28_H_23_F_3_N_4_S·CHCl_3_, the chloro­form solvate mol­ecules connect the pyrimidine mol­ecules into chains along [101] through weak C—H⋯N and C—H⋯Cl hydrogen-bond inter­actions. There are further connections between adjacent chains through F⋯Cl halogen contacts of 3.185 (3) Å, with the –CF_3_ group presenting a significant short F⋯F inter­chain distance of 2.712 (4) Å. The five-membered pyrazole ring is approximately planar (r.m.s. deviation = 0.050 Å). The pyrimidine ring makes dihedral angles of 84.15 (8) and 4.56 (8)° with the benzene rings.

## Related literature   

For the synthesis of the title compound and similar crystal structures, see: Flores *et al.* (2006 ▶). For biological properties of 4-tri­fluoro­methyl-2-(5-aryl-3-styryl-1*H*-pyrazol-1­yl)-pyrim­idines, see: Gressler *et al.* (2010 ▶). For halogen contacts, see: Baker *et al.* (2012 ▶); Metrangolo *et al.* (2008 ▶). For van der Waals radii, see: Batsanov (2001 ▶). For a related structure, see: Fabiani Claro Flores *et al.* (2014 ▶).
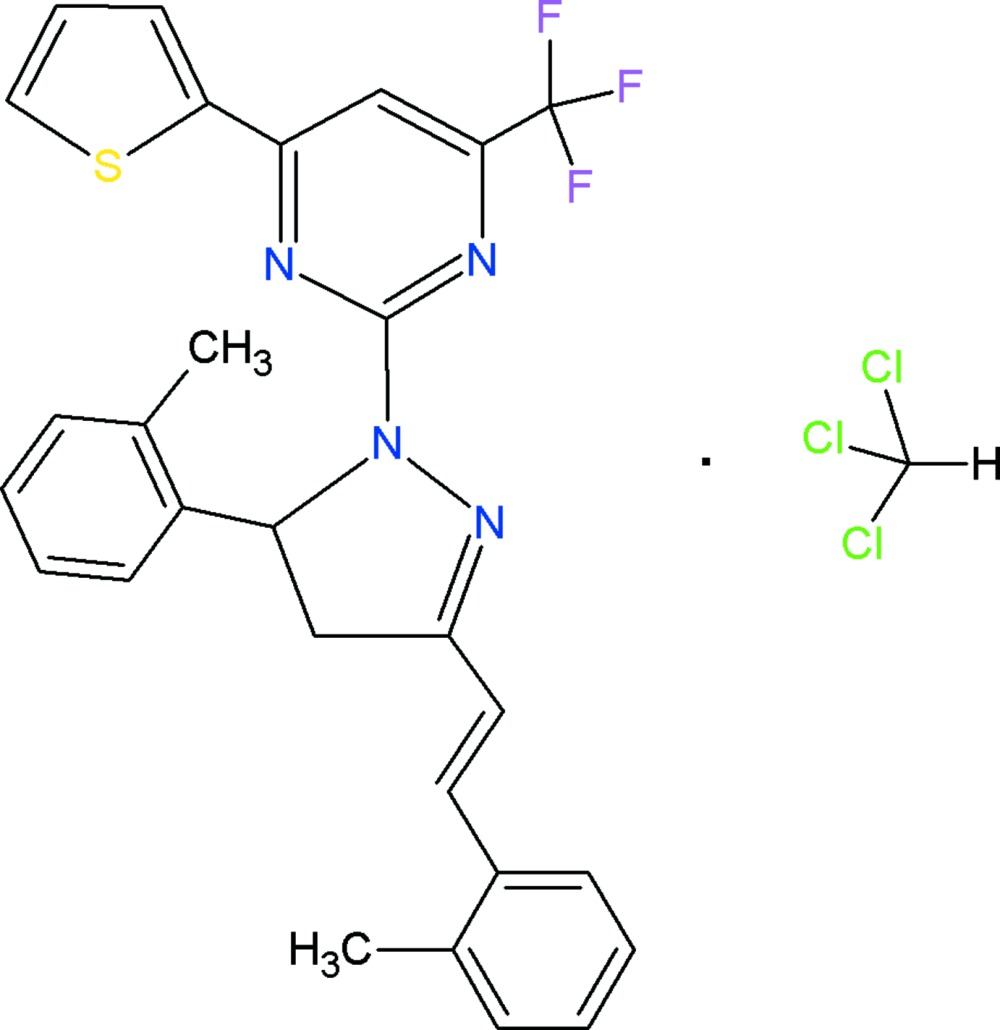


## Experimental   

### 

#### Crystal data  

C_28_H_23_F_3_N_4_S·CHCl_3_
*M* *_r_* = 623.93
Triclinic, 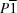

*a* = 10.6606 (3) Å
*b* = 11.0902 (3) Å
*c* = 13.4230 (4) Å
α = 100.518 (2)°
β = 105.863 (2)°
γ = 100.848 (2)°
*V* = 1452.55 (7) Å^3^
*Z* = 2
Mo *K*α radiation
μ = 0.43 mm^−1^
*T* = 273 K
0.31 × 0.28 × 0.16 mm

#### Data collection  

Bruker APEXII CCD diffractometer
Absorption correction: gaussian (*XPREP*; Bruker, 2009 ▶) *T*_min_ = 0.902, *T*_max_ = 1
45041 measured reflections
6974 independent reflections
4777 reflections with *I* > 2σ(*I*)
*R*_int_ = 0.024

#### Refinement  

*R*[*F* ^2^ > 2σ(*F* ^2^)] = 0.068
*wR*(*F* ^2^) = 0.242
*S* = 1.11
6974 reflections
361 parameters
H-atom parameters constrained
Δρ_max_ = 1.24 e Å^−3^
Δρ_min_ = −0.93 e Å^−3^

### 

Data collection: *APEX2* (Bruker, 2009 ▶); cell refinement: *SAINT* (Bruker, 2009 ▶); data reduction: *SAINT*; program(s) used to solve structure: *SHELXS97* (Sheldrick, 2008 ▶); program(s) used to refine structure: *SHELXL2013* (Sheldrick, 2008 ▶); molecular graphics: *DIAMOND* (Brandenburg, 2006 ▶); software used to prepare material for publication: *publCIF* (Westrip, 2010 ▶).

## Supplementary Material

Crystal structure: contains datablock(s) I. DOI: 10.1107/S160053681401321X/zq2224sup1.cif

Structure factors: contains datablock(s) I. DOI: 10.1107/S160053681401321X/zq2224Isup2.hkl

Supporting information file. DOI: 10.1107/S160053681401321X/zq2224Isup3.cml

CCDC reference: 1007013

Additional supporting information:  crystallographic information; 3D view; checkCIF report

## Figures and Tables

**Table 1:** Hydrogen-bond geometry (Å, °)

*D*—H⋯*A*	*D*—H	H⋯*A*	*D*⋯*A*	*D*—H⋯*A*
C20—H20⋯Cl1^i^	0.93	2.92	3.667 (3)	139
C29—H29⋯N2^i^	0.98	2.71	3.588 (4)	150
C1—H1⋯Cl1^ii^	0.93	2.91	3.606 (3)	133

Symmetry codes: (i) 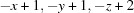; (ii) 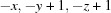.

**Fig. 1.**
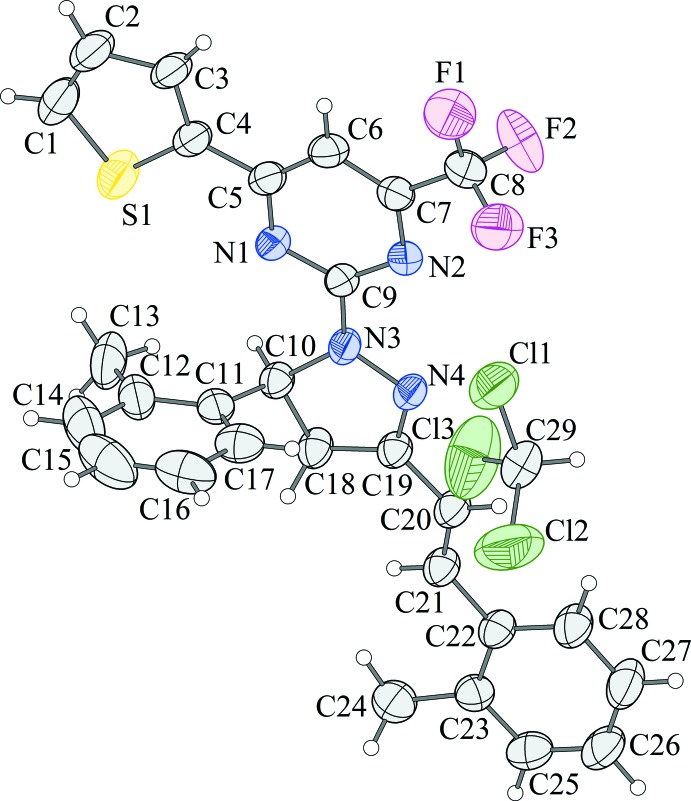
Asymmetric unit of the title compound. Ellipsoid probability: 50%.

**Fig. 2.**
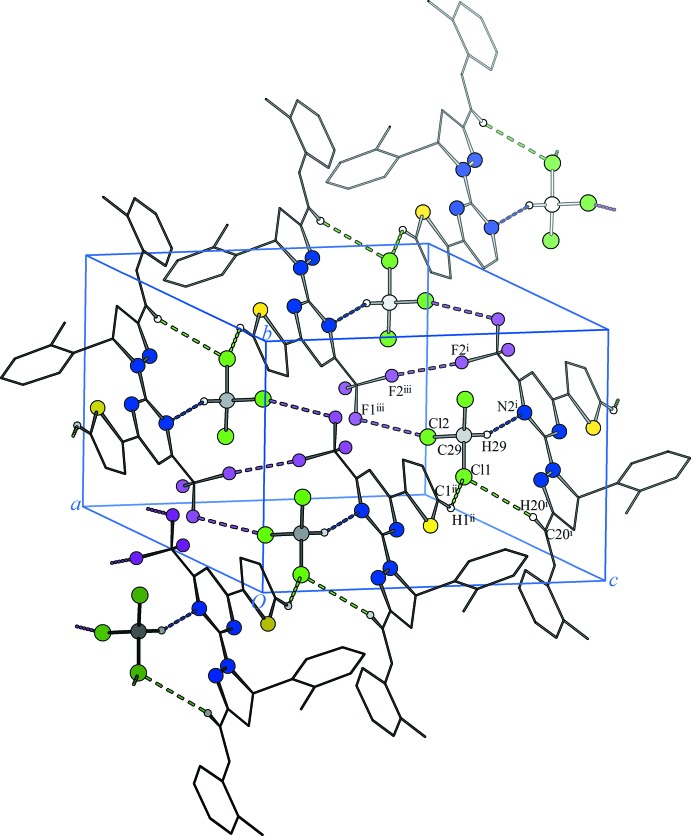
Packing diagram of the title compound showing polymer-like chains interacting through Cl3C—H···F, Cl2HC—Cl···F and F···F weak contacts represented as dashed lines. Most of the hydrogen atoms were omitted for clarity. Darker colors were used to emphasize front molecules. Symmetry codes: (i) –x + 1, –y + 1, –z + 2; (ii) –x, –y + 1, –z + 1; (iii) x + 1, y, z.

## supplementary crystallographic information

### S1. Comment

The 4-trifluoromethyl-2-(5-aryl-3-styryl-1*H*-pyrazol-1yl)-pyrimidines are biologically active compounds (Gressler *et al.*, 2010) obtained from a sequential two steps process involving [3 + 2] and [3 + 3] cyclocondensation starting from aminoguanidine (Flores *et al.*, 2006). In the crystal structure of the title compound, the asymmetric unit is composed by the whole organic molecule, including an additional chloroform solvate (Fig. 1) and displaying some interesting conformational features (Fabiani Claro Flores *et al.*, 2014). The styrene fragment is planar with r.m.s. deviation from the mean plane of 0.0456 Å and the least-squares plane angle between the 4,5-dihydropyrazole ring and the styrene fragment of 3.27 (19)°. Five-membered pyrazole and six-membered pyrimidine rings are also planar, with r.m.s. deviations from the plane of 0.0504 Å and 0.0044 Å, respectively. The torsion angle N4/N3/C9/N2 is 0.6 (4)°, showing that the pyrazole and pyrimidine rings are almost coplanar. The planarity can be confirmed by the pyrazole ring deviation from the least-squares plane by 1.87 (18)° from the pyrimidine ring. Additionally, the five-membered thien-2-yl ring is planar with r.m.s. deviation from the plane of 0.0040 Å and the least square plane angle between the pyrimidine ring and the thien-2-yl ring was 0.68 (18)°. This planarity observed is probably due to the π-resonance involving all system. The phenyl groupment C11/C12/C14/C15/C16/C17 deviates from the least-squares plane by 83.42 (9)° of 4,5-dihydropyrazole ring, indicating an orientation perpendicular between the rings. The geometry of the heterocyclic system is similar to that reported in the literature (Flores *et al.*, 2006). The chloroform solvate molecule plays an important role in the crystal structure packing, connecting molecules into polymer-like chains through the following weak hydrogen bond interactions (Fig. 2): C29—H29···N2^i^ (2.7056 (18) Å); C20^i^—H20^i^···Cl1 (2.9182 (12) Å); C1^ii^—H1^ii^···Cl1 (2.9072 (9) Å). The chloroform solvate also promotes further connections with adjacent chains through halogen long range contacts F1^iii^···Cl2 (3.1854 (29) Å). The CF_3_ group present a considerably short F2^iii^···F2^i^ distance of 2.712 (4) Å (Baker *et al.*, 2012), which may contribute to the arrangement observed in the solid state (symmetry codes: (i) –*x* + 1, –*y* + 1, –*z* + 2; (ii) –*x*, –*y* + 1, –*z* + 1; (iii) *x* + 1, *y*, *z*). All these interactions presented distances lesser than the sum of the van der Waals radii of the atoms involved (Batsanov, 2001). Besides, F···F contacts are quite similar to that described for larger halogen atoms, such as bromine and iodine (Metrangolo *et al.*, 2008).

### S2. Experimental

To a mixture of 1-carboxamidino-3-(2'-methylstyryl)-5-(2-methylphenyl)-4,5-dihydro-1*H*-pyrazole hydrochloride (1.2 mmol) (Flores *et al.*, 2006) and 1,1,1-trifluoro-4-methoxy-4-(tien-2-yl)-3-buten-2-one (1 mmol) in dry EtOH (3 ml) it was added three drops of BF_3_·OEt_2_, the mixture was stirred for 15 minutes. A yellow precipitated was isolated by filtration, washed with cold EtOH and recrystallized from CHCl_3_, affording the title compound.

### S3. Refinement

All H atoms attached to C atoms were positioned with idealized geometry and were refined isotropic with *U*_eq_(H) set to 1.5 times of the *U*_eq_(C) for CH_3_ groups and 1.2 otherwise. It was used a riding model with C—H = 0.96 Å for CH_3_, 0.97 Å for CH_2_, 0.98 Å for CH and 0.93 Å for aromatic CH. The following set of reflections was omitted due to the large difference observed between *F_o_*^2^ and Fc^2^: -1 -2 2; 0 0 1; 0 1 0; 1 0 0; -2 -3 4; -3 -3 1; -2 -3 3; -4 5 0; -6 -2 2; -2 -4 4; 2 -9 1; -2 8 0; 1 1 6; 1 1 4; 3 3 1.

### Crystal data

**Table:**

C_28_H_23_F_3_N_4_S·CHCl_3_	*Z* = 2
*M**_r_* = 623.93	*F*(000) = 640
Triclinic, *P*1	*D*_x_ = 1.427 Mg m^−^^3^
Hall symbol: -P 1	Mo *K*α radiation, λ = 0.71073 Å
*a* = 10.6606 (3) Å	Cell parameters from 6263 reflections
*b* = 11.0902 (3) Å	θ = 2.8–25.1°
*c* = 13.4230 (4) Å	µ = 0.43 mm^−^^1^
α = 100.518 (2)°	*T* = 273 K
β = 105.863 (2)°	Prismatic, colourless
γ = 100.848 (2)°	0.31 × 0.28 × 0.16 mm
*V* = 1452.55 (7) Å^3^	

### Data collection

**Table:**

Bruker APEXII CCD diffractometer	6974 independent reflections
Radiation source: fine-focus sealed tube	4777 reflections with *I* > 2σ(*I*)
Graphite monochromator	*R*_int_ = 0.024
*φ* and *ω* scans	θ_max_ = 28.0°, θ_min_ = 2.4°
Absorption correction: gaussian (*XPREP*; Bruker, 2009)	*h* = −14→14
*T*_min_ = 0.902, *T*_max_ = 1	*k* = −14→14
45041 measured reflections	*l* = −17→16

### Refinement

**Table:**

Refinement on *F*^2^	Primary atom site location: structure-invariant direct methods
Least-squares matrix: full	Secondary atom site location: difference Fourier map
*R*[*F*^2^ > 2σ(*F*^2^)] = 0.068	Hydrogen site location: inferred from neighbouring sites
*wR*(*F*^2^) = 0.242	H-atom parameters constrained
*S* = 1.11	*w* = 1/[σ^2^(*F*_o_^2^) + (0.1398*P*)^2^ + 0.5025*P*] where *P* = (*F*_o_^2^ + 2*F*_c_^2^)/3
6974 reflections	(Δ/σ)_max_ < 0.001
361 parameters	Δρ_max_ = 1.24 e Å^−^^3^
0 restraints	Δρ_min_ = −0.93 e Å^−^^3^

### Special details

**Table:**

Experimental. Absorption correction: XPREP (Bruker, 2009) was used to perform the Gaussian absorption correction based on the face-indexed crystal size.
Geometry. All e.s.d.'s (except the e.s.d. in the dihedral angle between two l.s. planes) are estimated using the full covariance matrix. The cell e.s.d.'s are taken into account individually in the estimation of e.s.d.'s in distances, angles and torsion angles; correlations between e.s.d.'s in cell parameters are only used when they are defined by crystal symmetry. An approximate (isotropic) treatment of cell e.s.d.'s is used for estimating e.s.d.'s involving l.s. planes.
Refinement. Refinement of *F*^2^ against ALL reflections. The weighted *R*-factor *wR* and goodness of fit *S* are based on *F*^2^, conventional *R*-factors *R* are based on *F*, with *F* set to zero for negative *F*^2^. The threshold expression of *F*^2^ > σ(*F*^2^) is used only for calculating *R*-factors(gt) *etc*. and is not relevant to the choice of reflections for refinement. *R*-factors based on *F*^2^ are statistically about twice as large as those based on *F*, and *R*- factors based on ALL data will be even larger.

### Fractional atomic coordinates and isotropic or equivalent isotropic displacement parameters (Å^2^)

**Table:**

	*x*	*y*	*z*	*U*_iso_*/*U*_eq_	
S1	−0.01728 (9)	0.76442 (8)	0.50222 (7)	0.0682 (3)	
C11	0.4130 (2)	0.8288 (2)	0.65685 (19)	0.0438 (6)	
C17	0.4753 (3)	0.7295 (3)	0.6497 (3)	0.0572 (7)	
H17	0.5028	0.6966	0.7087	0.069*	
C12	0.3698 (3)	0.8789 (3)	0.5683 (2)	0.0595 (8)	
C16	0.4966 (4)	0.6790 (4)	0.5550 (3)	0.0776 (11)	
H16	0.5410	0.6145	0.5511	0.093*	
C15	0.4518 (5)	0.7249 (5)	0.4671 (4)	0.0985 (16)	
H15	0.4633	0.6896	0.4029	0.118*	
C14	0.3895 (5)	0.8234 (4)	0.4737 (3)	0.0860 (12)	
H14	0.3600	0.8535	0.4134	0.103*	
Cl1	0.44264 (9)	0.26973 (10)	0.81760 (8)	0.0848 (3)	
Cl3	0.5764 (2)	0.52932 (12)	0.88980 (12)	0.1361 (7)	
Cl2	0.72237 (12)	0.3306 (2)	0.85929 (13)	0.1408 (7)	
C29	0.5949 (3)	0.3790 (3)	0.8970 (3)	0.0664 (8)	
H29	0.6084	0.3740	0.9712	0.080*	
C23	1.0217 (3)	1.1438 (2)	1.1310 (2)	0.0462 (6)	
C22	0.9161 (2)	1.0415 (2)	1.12276 (19)	0.0435 (6)	
C28	0.9355 (3)	0.9708 (3)	1.1992 (2)	0.0593 (8)	
H28	0.8649	0.9046	1.1954	0.071*	
C25	1.1422 (3)	1.1681 (3)	1.2150 (3)	0.0618 (8)	
H25	1.2126	1.2360	1.2215	0.074*	
C26	1.1608 (3)	1.0965 (3)	1.2874 (3)	0.0679 (9)	
H26	1.2428	1.1143	1.3414	0.081*	
C27	1.0552 (4)	0.9959 (3)	1.2795 (3)	0.0699 (9)	
H27	1.0659	0.9462	1.3285	0.084*	
N2	0.22412 (19)	0.6049 (2)	0.83093 (15)	0.0411 (5)	
N1	0.15480 (19)	0.71151 (19)	0.69225 (15)	0.0395 (4)	
C9	0.2431 (2)	0.6973 (2)	0.78029 (18)	0.0377 (5)	
C6	0.0076 (2)	0.5238 (3)	0.6959 (2)	0.0467 (6)	
H6	−0.0739	0.4625	0.6668	0.056*	
C5	0.0375 (2)	0.6247 (2)	0.65126 (18)	0.0409 (5)	
C7	0.1059 (2)	0.5198 (2)	0.78595 (19)	0.0413 (5)	
N4	0.4644 (2)	0.7868 (2)	0.90721 (15)	0.0431 (5)	
N3	0.3620 (2)	0.7876 (2)	0.81858 (15)	0.0423 (5)	
C10	0.3995 (2)	0.8858 (2)	0.76382 (18)	0.0401 (5)	
H10	0.3334	0.9369	0.7545	0.048*	
C19	0.5630 (2)	0.8833 (2)	0.92339 (18)	0.0404 (5)	
C21	0.7875 (2)	1.0104 (2)	1.03551 (19)	0.0454 (6)	
H21	0.7757	1.0682	0.9936	0.055*	
C18	0.5347 (3)	0.9649 (2)	0.84646 (19)	0.0448 (6)	
H18B	0.6048	0.9786	0.8134	0.054*	
H18A	0.5266	1.0463	0.8816	0.054*	
C20	0.6865 (2)	0.9077 (3)	1.0108 (2)	0.0463 (6)	
H20	0.6959	0.8488	1.0517	0.056*	
C3	−0.1863 (3)	0.5594 (3)	0.4960 (2)	0.0534 (7)	
H3	−0.2261	0.4863	0.5118	0.064*	
C4	−0.0573 (2)	0.6396 (3)	0.5552 (2)	0.0447 (6)	
C1	−0.1672 (3)	0.7167 (4)	0.4033 (3)	0.0666 (8)	
H1	−0.1924	0.7605	0.3513	0.080*	
C2	−0.2467 (3)	0.6086 (4)	0.4072 (2)	0.0654 (9)	
H2	−0.3315	0.5699	0.3578	0.078*	
C8	0.0840 (3)	0.4152 (3)	0.8404 (2)	0.0531 (7)	
F1	−0.0216 (2)	0.32016 (19)	0.78234 (18)	0.0835 (6)	
F3	0.1888 (2)	0.3649 (2)	0.8628 (2)	0.0819 (6)	
F2	0.0635 (3)	0.4554 (2)	0.93190 (18)	0.0898 (7)	
C24	1.0106 (3)	1.2256 (3)	1.0535 (3)	0.0641 (8)	
H24C	0.9226	1.1971	1.0010	0.096*	
H24B	1.0777	1.2202	1.0186	0.096*	
H24A	1.0244	1.3118	1.0910	0.096*	
C13	0.3074 (4)	0.9893 (4)	0.5745 (3)	0.0785 (10)	
H13A	0.3042	1.0170	0.6456	0.118*	
H13C	0.2176	0.9642	0.5248	0.118*	
H13B	0.3603	1.0574	0.5570	0.118*	

### Atomic displacement parameters (Å^2^)

**Table:**

	*U*^11^	*U*^22^	*U*^33^	*U*^12^	*U*^13^	*U*^23^
S1	0.0641 (5)	0.0673 (5)	0.0585 (5)	0.0090 (4)	−0.0020 (3)	0.0201 (4)
C11	0.0383 (11)	0.0434 (13)	0.0392 (12)	−0.0038 (10)	0.0074 (9)	0.0071 (10)
C17	0.0474 (14)	0.0498 (16)	0.0626 (17)	0.0020 (12)	0.0155 (12)	−0.0011 (13)
C12	0.0619 (16)	0.0635 (18)	0.0427 (14)	−0.0045 (14)	0.0108 (12)	0.0174 (12)
C16	0.067 (2)	0.067 (2)	0.088 (3)	0.0004 (16)	0.0387 (19)	−0.0115 (19)
C15	0.109 (3)	0.100 (3)	0.069 (3)	−0.018 (3)	0.053 (2)	−0.015 (2)
C14	0.107 (3)	0.093 (3)	0.0460 (18)	−0.004 (2)	0.0272 (19)	0.0144 (18)
Cl1	0.0610 (5)	0.0950 (7)	0.0881 (6)	0.0108 (4)	0.0046 (4)	0.0360 (5)
Cl3	0.1896 (16)	0.0734 (7)	0.1109 (10)	0.0221 (8)	−0.0054 (10)	0.0323 (7)
Cl2	0.0650 (7)	0.235 (2)	0.1214 (11)	0.0516 (9)	0.0312 (7)	0.0244 (11)
C29	0.0627 (18)	0.077 (2)	0.0523 (16)	0.0201 (16)	0.0041 (13)	0.0185 (15)
C23	0.0416 (12)	0.0459 (14)	0.0461 (13)	0.0052 (10)	0.0116 (10)	0.0092 (11)
C22	0.0417 (12)	0.0403 (13)	0.0386 (12)	0.0052 (10)	0.0040 (9)	0.0043 (10)
C28	0.0522 (15)	0.0561 (17)	0.0515 (15)	−0.0062 (12)	−0.0020 (12)	0.0184 (13)
C25	0.0411 (13)	0.0646 (19)	0.0643 (18)	−0.0034 (12)	0.0054 (12)	0.0129 (14)
C26	0.0485 (15)	0.078 (2)	0.0551 (16)	0.0014 (14)	−0.0072 (12)	0.0148 (15)
C27	0.0674 (19)	0.072 (2)	0.0509 (16)	0.0020 (16)	−0.0063 (14)	0.0232 (15)
N2	0.0359 (9)	0.0469 (11)	0.0386 (10)	0.0062 (8)	0.0106 (8)	0.0127 (8)
N1	0.0350 (9)	0.0444 (11)	0.0359 (10)	0.0091 (8)	0.0074 (7)	0.0091 (8)
C9	0.0331 (10)	0.0429 (13)	0.0353 (11)	0.0082 (9)	0.0094 (8)	0.0090 (9)
C6	0.0347 (11)	0.0500 (15)	0.0501 (14)	0.0048 (10)	0.0108 (10)	0.0100 (11)
C5	0.0334 (10)	0.0484 (14)	0.0386 (12)	0.0121 (10)	0.0095 (9)	0.0061 (10)
C7	0.0385 (11)	0.0435 (13)	0.0427 (12)	0.0082 (10)	0.0163 (9)	0.0098 (10)
N4	0.0362 (9)	0.0495 (12)	0.0373 (10)	0.0053 (8)	0.0027 (8)	0.0145 (9)
N3	0.0380 (10)	0.0461 (12)	0.0369 (10)	0.0039 (8)	0.0035 (8)	0.0157 (8)
C10	0.0388 (11)	0.0372 (12)	0.0397 (12)	0.0055 (9)	0.0069 (9)	0.0110 (9)
C19	0.0390 (11)	0.0403 (12)	0.0356 (11)	0.0060 (9)	0.0061 (9)	0.0069 (9)
C21	0.0434 (12)	0.0440 (13)	0.0399 (12)	0.0053 (10)	0.0039 (10)	0.0085 (10)
C18	0.0475 (13)	0.0388 (13)	0.0379 (12)	0.0027 (10)	0.0033 (10)	0.0087 (10)
C20	0.0408 (12)	0.0474 (14)	0.0424 (12)	0.0048 (10)	0.0031 (10)	0.0123 (10)
C3	0.0343 (12)	0.0724 (19)	0.0447 (13)	0.0144 (12)	0.0015 (10)	0.0088 (12)
C4	0.0360 (11)	0.0522 (15)	0.0420 (12)	0.0133 (10)	0.0070 (9)	0.0078 (11)
C1	0.0600 (17)	0.082 (2)	0.0528 (16)	0.0280 (17)	0.0008 (13)	0.0196 (15)
C2	0.0439 (14)	0.089 (2)	0.0522 (16)	0.0213 (15)	−0.0012 (12)	0.0114 (15)
C8	0.0473 (14)	0.0529 (16)	0.0582 (16)	0.0045 (12)	0.0189 (12)	0.0168 (13)
F1	0.0741 (13)	0.0630 (12)	0.0957 (15)	−0.0129 (10)	0.0130 (11)	0.0288 (11)
F3	0.0684 (12)	0.0774 (13)	0.1198 (17)	0.0293 (10)	0.0324 (12)	0.0574 (12)
F2	0.139 (2)	0.0786 (14)	0.0794 (14)	0.0263 (14)	0.0685 (14)	0.0346 (11)
C24	0.0557 (16)	0.0616 (18)	0.0724 (19)	0.0038 (14)	0.0174 (14)	0.0262 (15)
C13	0.093 (3)	0.077 (2)	0.0596 (19)	0.0149 (19)	0.0050 (17)	0.0383 (17)

### Geometric parameters (Å, º)

**Table:**

S1—C1	1.692 (3)	C9—N3	1.361 (3)
S1—C4	1.705 (3)	C6—C7	1.381 (3)
C11—C17	1.391 (4)	C6—C5	1.388 (4)
C11—C12	1.403 (4)	C6—H6	0.9300
C11—C10	1.516 (4)	C5—C4	1.463 (3)
C17—C16	1.388 (5)	C7—C8	1.494 (4)
C17—H17	0.9300	N4—C19	1.289 (3)
C12—C14	1.395 (5)	N4—N3	1.380 (3)
C12—C13	1.500 (5)	N3—C10	1.475 (3)
C16—C15	1.371 (7)	C10—C18	1.541 (3)
C16—H16	0.9300	C10—H10	0.9800
C15—C14	1.383 (7)	C19—C20	1.445 (3)
C15—H15	0.9300	C19—C18	1.500 (3)
C14—H14	0.9300	C21—C20	1.331 (4)
Cl1—C29	1.755 (4)	C21—H21	0.9300
Cl3—C29	1.732 (4)	C18—H18B	0.9700
Cl2—C29	1.716 (4)	C18—H18A	0.9700
C29—H29	0.9800	C20—H20	0.9300
C23—C25	1.401 (4)	C3—C4	1.413 (4)
C23—C22	1.405 (4)	C3—C2	1.444 (4)
C23—C24	1.495 (4)	C3—H3	0.9300
C22—C28	1.396 (4)	C1—C2	1.350 (5)
C22—C21	1.472 (3)	C1—H1	0.9300
C28—C27	1.368 (4)	C2—H2	0.9300
C28—H28	0.9300	C8—F2	1.319 (4)
C25—C26	1.361 (5)	C8—F3	1.327 (4)
C25—H25	0.9300	C8—F1	1.327 (3)
C26—C27	1.393 (5)	C24—H24C	0.9600
C26—H26	0.9300	C24—H24B	0.9600
C27—H27	0.9300	C24—H24A	0.9600
N2—C7	1.331 (3)	C13—H13A	0.9600
N2—C9	1.343 (3)	C13—H13C	0.9600
N1—C5	1.330 (3)	C13—H13B	0.9600
N1—C9	1.350 (3)		
			
C1—S1—C4	91.99 (16)	C6—C7—C8	120.5 (2)
C17—C11—C12	120.6 (3)	C19—N4—N3	107.67 (19)
C17—C11—C10	118.4 (2)	C9—N3—N4	122.22 (19)
C12—C11—C10	120.9 (3)	C9—N3—C10	123.73 (18)
C16—C17—C11	120.5 (3)	N4—N3—C10	113.73 (18)
C16—C17—H17	119.8	N3—C10—C11	111.8 (2)
C11—C17—H17	119.8	N3—C10—C18	100.66 (18)
C14—C12—C11	117.3 (4)	C11—C10—C18	113.0 (2)
C14—C12—C13	120.6 (3)	N3—C10—H10	110.4
C11—C12—C13	122.0 (3)	C11—C10—H10	110.4
C15—C16—C17	119.5 (4)	C18—C10—H10	110.4
C15—C16—H16	120.2	N4—C19—C20	120.8 (2)
C17—C16—H16	120.2	N4—C19—C18	114.1 (2)
C16—C15—C14	120.3 (3)	C20—C19—C18	125.1 (2)
C16—C15—H15	119.9	C20—C21—C22	126.3 (2)
C14—C15—H15	119.9	C20—C21—H21	116.9
C15—C14—C12	121.8 (4)	C22—C21—H21	116.9
C15—C14—H14	119.1	C19—C18—C10	102.5 (2)
C12—C14—H14	119.1	C19—C18—H18B	111.3
Cl2—C29—Cl3	118.3 (2)	C10—C18—H18B	111.3
Cl2—C29—Cl1	107.7 (2)	C19—C18—H18A	111.3
Cl3—C29—Cl1	107.82 (18)	C10—C18—H18A	111.3
Cl2—C29—H29	107.6	H18B—C18—H18A	109.2
Cl3—C29—H29	107.6	C21—C20—C19	123.2 (2)
Cl1—C29—H29	107.6	C21—C20—H20	118.4
C25—C23—C22	117.9 (3)	C19—C20—H20	118.4
C25—C23—C24	119.4 (3)	C4—C3—C2	109.4 (3)
C22—C23—C24	122.7 (2)	C4—C3—H3	125.3
C28—C22—C23	118.7 (2)	C2—C3—H3	125.3
C28—C22—C21	120.9 (2)	C3—C4—C5	127.3 (3)
C23—C22—C21	120.4 (2)	C3—C4—S1	112.3 (2)
C27—C28—C22	122.0 (3)	C5—C4—S1	120.49 (19)
C27—C28—H28	119.0	C2—C1—S1	113.3 (2)
C22—C28—H28	119.0	C2—C1—H1	123.4
C26—C25—C23	122.8 (3)	S1—C1—H1	123.4
C26—C25—H25	118.6	C1—C2—C3	113.0 (3)
C23—C25—H25	118.6	C1—C2—H2	123.5
C25—C26—C27	119.0 (3)	C3—C2—H2	123.5
C25—C26—H26	120.5	F2—C8—F3	106.5 (3)
C27—C26—H26	120.5	F2—C8—F1	106.5 (2)
C28—C27—C26	119.6 (3)	F3—C8—F1	106.3 (2)
C28—C27—H27	120.2	F2—C8—C7	111.6 (2)
C26—C27—H27	120.2	F3—C8—C7	112.5 (2)
C7—N2—C9	114.4 (2)	F1—C8—C7	113.0 (2)
C5—N1—C9	116.6 (2)	C23—C24—H24C	109.5
N2—C9—N1	126.6 (2)	C23—C24—H24B	109.5
N2—C9—N3	119.0 (2)	H24C—C24—H24B	109.5
N1—C9—N3	114.4 (2)	C23—C24—H24A	109.5
C7—C6—C5	116.1 (2)	H24C—C24—H24A	109.5
C7—C6—H6	122.0	H24B—C24—H24A	109.5
C5—C6—H6	122.0	C12—C13—H13A	109.5
N1—C5—C6	121.9 (2)	C12—C13—H13C	109.5
N1—C5—C4	116.5 (2)	H13A—C13—H13C	109.5
C6—C5—C4	121.7 (2)	C12—C13—H13B	109.5
N2—C7—C6	124.5 (2)	H13A—C13—H13B	109.5
N2—C7—C8	115.1 (2)	H13C—C13—H13B	109.5
			
C12—C11—C17—C16	0.6 (4)	C19—N4—N3—C9	179.9 (2)
C10—C11—C17—C16	−176.4 (3)	C19—N4—N3—C10	6.2 (3)
C17—C11—C12—C14	1.2 (4)	C9—N3—C10—C11	−64.4 (3)
C10—C11—C12—C14	178.1 (3)	N4—N3—C10—C11	109.2 (2)
C17—C11—C12—C13	−177.5 (3)	C9—N3—C10—C18	175.5 (2)
C10—C11—C12—C13	−0.6 (4)	N4—N3—C10—C18	−10.9 (3)
C11—C17—C16—C15	−2.2 (5)	C17—C11—C10—N3	−41.0 (3)
C17—C16—C15—C14	2.0 (6)	C12—C11—C10—N3	142.0 (2)
C16—C15—C14—C12	−0.2 (6)	C17—C11—C10—C18	71.7 (3)
C11—C12—C14—C15	−1.4 (5)	C12—C11—C10—C18	−105.3 (3)
C13—C12—C14—C15	177.3 (4)	N3—N4—C19—C20	−179.4 (2)
C25—C23—C22—C28	1.1 (4)	N3—N4—C19—C18	1.9 (3)
C24—C23—C22—C28	−179.7 (3)	C28—C22—C21—C20	−9.4 (5)
C25—C23—C22—C21	−179.3 (3)	C23—C22—C21—C20	171.0 (3)
C24—C23—C22—C21	−0.1 (4)	N4—C19—C18—C10	−8.5 (3)
C23—C22—C28—C27	−2.0 (5)	C20—C19—C18—C10	172.9 (2)
C21—C22—C28—C27	178.4 (3)	N3—C10—C18—C19	10.6 (2)
C22—C23—C25—C26	0.5 (5)	C11—C10—C18—C19	−108.7 (2)
C24—C23—C25—C26	−178.7 (3)	C22—C21—C20—C19	−179.7 (2)
C23—C25—C26—C27	−1.2 (6)	N4—C19—C20—C21	−175.2 (3)
C22—C28—C27—C26	1.3 (6)	C18—C19—C20—C21	3.3 (4)
C25—C26—C27—C28	0.3 (6)	C2—C3—C4—C5	179.8 (3)
C7—N2—C9—N1	1.2 (4)	C2—C3—C4—S1	−0.3 (3)
C7—N2—C9—N3	−178.6 (2)	N1—C5—C4—C3	178.8 (2)
C5—N1—C9—N2	−0.4 (4)	C6—C5—C4—C3	0.1 (4)
C5—N1—C9—N3	179.3 (2)	N1—C5—C4—S1	−1.1 (3)
C9—N1—C5—C6	−0.6 (3)	C6—C5—C4—S1	−179.9 (2)
C9—N1—C5—C4	−179.3 (2)	C1—S1—C4—C3	0.6 (2)
C7—C6—C5—N1	0.8 (4)	C1—S1—C4—C5	−179.4 (2)
C7—C6—C5—C4	179.4 (2)	C4—S1—C1—C2	−0.9 (3)
C9—N2—C7—C6	−1.0 (4)	S1—C1—C2—C3	0.9 (4)
C9—N2—C7—C8	179.1 (2)	C4—C3—C2—C1	−0.4 (4)
C5—C6—C7—N2	0.1 (4)	N2—C7—C8—F2	72.4 (3)
C5—C6—C7—C8	180.0 (2)	C6—C7—C8—F2	−107.5 (3)
N2—C9—N3—N4	0.6 (4)	N2—C7—C8—F3	−47.2 (3)
N1—C9—N3—N4	−179.2 (2)	C6—C7—C8—F3	132.9 (3)
N2—C9—N3—C10	173.7 (2)	N2—C7—C8—F1	−167.5 (2)
N1—C9—N3—C10	−6.1 (3)	C6—C7—C8—F1	12.6 (4)

### Hydrogen-bond geometry (Å, º)

**Table:**

*D*—H···*A*	*D*—H	H···*A*	*D*···*A*	*D*—H···*A*
C20—H20···Cl1^i^	0.93	2.92	3.667 (3)	139
C29—H29···N2^i^	0.98	2.71	3.588 (4)	150
C1—H1···Cl1^ii^	0.93	2.91	3.606 (3)	133

Symmetry codes: (i) −*x*+1, −*y*+1, −*z*+2; (ii) −*x*, −*y*+1, −*z*+1.
